# Fusion versus Nonfusion for Surgically Treated Thoracolumbar Burst Fractures: A Meta-Analysis

**DOI:** 10.1371/journal.pone.0063995

**Published:** 2013-05-21

**Authors:** Nai-Feng Tian, Yao-Sen Wu, Xiao-Lei Zhang, Xin-Lei Wu, Yong-Long Chi, Fang-Min Mao

**Affiliations:** 1 Department of Orthopaedic Surgery, Second Affiliated Hospital of Wenzhou Medical College, Wenzhou, China; 2 Department of Orthopaedics, Second Affiliated Hospital, School of Medicine, Zhejiang University, Hangzhou, China; 3 Center for Stem Cells and Tissue Engineering, School of Medicine, Zhejiang University, Hangzhou, China; 4 Institute of Digitized Medicine, Wenzhou Medical College, Wenzhou, China; Universität München, Germany

## Abstract

**Background:**

Posterior pedicle screw fixation has become a popular method for treating thoracolumbar burst fractures. However, it remains unclear whether additional fusion could improve clinical and radiological outcomes. This meta-analysis was performed to evaluate the effectiveness of fusion as a supplement to pedicle screw fixation for thoracolumbar burst fractures.

**Methodology/Principal Findings:**

MEDLINE, OVID, Springer, and Google Scholar were searched for relevant randomized and quasi-randomized controlled trials that compared the clinical and radiological efficacy of fusion versus nonfusion for thoracolumbar burst fractures managed by posterior pedicle screw fixation. Risk of bias in included studies was assessed using the Cochrane Risk of Bias tool. We generated pooled risk ratios or weighted mean differences across studies. Based on predefined inclusion criteria, 4 eligible trials with a total of 220 patients were included in this meta-analysis. The mean age of the patients was 35.1 years. 96.8% of the fractures were located at T12 to L1 level. Baseline characteristics were similar between the fusion and nonfusion groups. No significant difference was identified between the two groups regarding radiological outcome, functional outcome, neurologic improvement, and implant failure rate. The pooled data showed that the nonfusion group was associated with significantly reduced operative time (p<0.0001) and blood loss (p  = 0.0003).

**Conclusions/Significances:**

The results of this meta-analysis suggested that fusion was not necessary when thoracolumbar burst fracture was treated by posterior pedicle screw fixation. More randomized controlled trials with high quality are still needed in the future.

## Introduction

Spinal “burst fracture”, firstly described by Holdsworth et al. [1], was redefined by Denis et al. [2] in 1983. According to Denis classification, burst fracture refers to failure of at least the anterior and middle columns of the spine [2]. They account for a quarter to half of all fractures in the thoracolumbar region where the majority of spinal fractures occur [3], [4]. Thoracolumbar burst fractures, frequently associated with kyphotic deformity and neurological deficit, are very common in younger patients and could have a great impact on their daily physical activities [4]. However, there is still controversy regarding the ideal management for these injuries.

Nonoperative treatment has been advocated for thoracolumbar burst fractures because it has the advantage of avoiding many surgical related complications [3], [5]–[7]. Some studies have revealed that conservative treatment could lead to satisfactory outcomes particularly in patients without neurological deficit [5]–[7]. However, lost correction of vertebral body height or kyphotic angle was often seen in these patients [7]. Surgical treatment for thoracolumbar burst fractures was intended to restore vertebral height, correct kyphosis, decompress neurostructures, and maintain stability [3]. It allowed early ambulation and rehabilitation, thus facilitating nursing care and avoiding complications associated with prolonged bed rest [3]. Posterior pedicle screw fixation has become a popular method for thoracolumbar burst fractures as it provides three-column stabilization. Numerous studies have proved the clinical and radiological efficiency of using this technique [4], [8]–[13]. In order to augment fixation strength, posterior or posterolateral fusion was frequently used [10]. Nevertheless, favorable clinical outcomes were also reported using posterior fixation without fusion [9]. Recently, several trials have been conducted comparing the clinical and radiological efficacy of posterior fixation with fusion versus posterior fixation without fusion [14]–[18]. The objective of this meta-analysis was to identify and summarize the evidence from randomized controlled trials (RCTs) on the effectiveness of fusion as a supplement to posterior instrumentation for thoracolumbar burst fractures.

## Methods

### Search Strategy and Selection Criteria

This meta-analysis was performed in accordance with the PRISMA Statement (Checklist S1). A systematic literature search was conducted up to September, 2012 using MEDLINE, OVID, Springer, and Google Scholar. We screened the title and abstract by combining the term “fusion”, or “nonfusion” with each of the following keywords: “thoracic fracture(s)”, “lumbar fracture(s)”, “thoracolumbar fracture(s)”, or “burst fracture(s)”. There was no language restriction. A comprehensive search of reference lists of published articles was also performed to ensure inclusion of all possible studies. Unpublished data were not reviewed. The following eligibility criteria were applied: (1) randomized or quasi-randomized (eg: ‘randomized’ by date of birth, hospital record number, or alternation) controlled trial. (2) The study compared the clinical and radiological outcomes of posterior fixation with fusion versus posterior fixation without fusion for thoracolumbar or lumbar burst fractures. (3) Burst fractures were confirmed through radiographs or computed tomographic scans. (4) Adult patients (≥18 years). (5) Over twelve-month follow-up. Patients with neurological deficit were not excluded. All potential records selected by the search strategy were independently reviewed by two investigators for inclusion into the final analysis. Inconsistencies were resolved through discussion until a consensus was reached.

### Data Extraction

Two reviewers independently extracted data using a standardized form. Data were collected based on following categories where available. (1) Basic characteristics, including published year, study design, inclusion/exclusion criteria, age, sex, enrolled number, and follow-up rate. (2) Injury information, consisting of classification and location of fracture, neurological status, preoperative spinal canal compromise, preoperative kyphotic angle, preoperative decreased vertebral body height (VBH), and time of injury to surgery. (3) Surgical information, such as fixed levels (short or long segment fixation), type of internal fixation, type of bone graft, fusion site, and spinal canal decompression information. (4) Primary outcomes, including correction of kyphotic angle, correction of decreased VBH, functional outcome, neurological improvement, and complications. (5) Secondary outcomes, consisting of operative time, blood loss, and hospital stay. Any disagreement between the reviewers was resolved by discussion.

### Risk of Bias Assessment

We assessed the risk of bias using the criteria according to Cochrane Handbook for Systematic Reviews of Interventions. Seven domains were assessed in each included studies. (1) Random sequence generation. (2) Allocation concealment. (3) Blinding of participants and personnel. (4) Blinding of outcome assessment. (5) Incomplete outcome data. (6) Selective reporting. (7) Other bias. Reviewers’ judgments were categorized as “low risk” of bias, “high risk” of bias or “unclear risk” of bias.

### Statistical Analysis

Both baseline characteristics and surgical outcomes were pooled and analyzed. Meta-analysis was performed using Review Manager 5.0 software (Cochrane Collaboration, Oxford, UK). Risk ratios (RR) were calculated for binary outcomes and weighted mean differences (WMD) for continuous outcomes, along with 95% confidence intervals (CI). The level of significance was set at p<0.05. Heterogeneity was evaluated using the χ^2^ test and I^2^ statistics (considered significant when p value for χ^2^ test <0.10 or I^2^>50%). Fixed-effect models were applied unless statistical heterogeneity was significant, in which case random-effect models were used. Through subgroup analysis, we investigated the influence of study design (RCT or quasi-RCT) and fixed levels (short or long segment fixation) on pooled estimates.

## Results

### Literature Search

The search strategy (Figure S1) identified 351 potential studies from the databases. 347 papers were excluded according to our inclusion criteria. No additional studies were obtained after reference review. Finally, four trials including three RCTs and one quasi-RCT were selected and analyzed [15]–[18]. Three RCTs assessed the impact of fusion as a supplement to short-segment fixation (one level above and one level below the injured segment) for thoracolumbar burst fractures [15], [16], [18]. The quasi-RCT investigated the role of fusion after long-segment instrumentation (two levels above and two levels below the injured segment) [17].

### Risk of Bias Assessment

Three trials described adequate methods of random sequence generation [15], [16], [18]. In the quasi-RCT, patients were allocated according to sequence of hospitalization [17]. Information of allocation concealment was not available in any of the studies. Due to the nature of the trials, it was impossible to perform blinding of participants and personnel. Two studies [16], [18] reported blinding of outcome assessment while the other two [15], [17] did not. In three trials, no patients were lost to follow-up after randomized allocation [15]–[17]. In Jindal’s study [18], three patients were excluded based on pre-defined criteria. Since the missing data was small in number, which also balanced in both arms, we regarded it as low risk of bias of incomplete outcome data addressed. In all trials, the outcomes were provided in detail. We considered them with a low risk of bias of selective reporting. Owing to insufficient information to assess whether an important risk of bias existed in a number of trials, we argued all trials had unclear risk of bias towards other potential sources of bias. The methodological quality assessment was summarized in Table 1.

**Table 1:** Risk of bias assessment of all included studies.

Risk of bias assessment	Wang 2006	Dai 2009	Jindal 2012	Tezeren 2009
Random sequence generation	Low risk	Low risk	Low risk	High risk
Allocation concealment	Unclear risk	Unclear risk	Unclear risk	Unclear risk
Blinding of participants and personnel	High risk	High risk	High risk	High risk
Blinding of outcome assessment	Unclear risk	Low risk	Low risk	Unclear risk
Incomplete outcome data addressed	Low risk	Low risk	Low risk	Low risk
Selective reporting	Low risk	Low risk	Low risk	Low risk
Free of other bias	Unclear risk	Unclear risk	Unclear risk	Unclear risk

### Study Characteristics

The basic information of the four included studies was presented in Table S1. A total of 220 patients with male to female ratio of 2.5∶1 were evaluated. The mean age was 35.1 years. The mean duration of follow-up ranged from 23.9 to 72 months. 96.8% of the fractures were located at T12 to L1 level. One study enrolled only neurological intact patients [17]. Neurological deficit wasn’t the exclusion criteria in the other three trials [15], [16], [18]. Preoperative spinal canal comprise and vertebral body height decrease were assessed in two studies [15], [17]. Three papers provided information about preoperative kyphotic angle and injury to surgery time [15], [16], [18]. Short segment pedicle screw fixation was applied in the three RCTs [15], [16], [18]. The quasi-RCT used long segment fixation through pedicle screws and hooks [17]. Autogenous iliac crest bone graft (AICBG) was used in all patients for posterior and/or posterolateral fusion [15]–[18]. The main complications reported in the included studies were implant failure and donor site pain. Only one patient experienced superficial infection. Statistically similar baseline characteristics were observed between the fusion and nonfusion groups (Table 2).

**Table 2:** Comparison of baseline characteristics between the fusion and nonfusion groups.

Characteristic	Wang 2006	Dai 2009	Jindal 2012	Tezeren 2009	All studies
Mean age	*	*	*	*	*
Gender	*	*	*	*	*
Follow-up time	*	*	*	*	*
Location of fracture	0.023	*	*	*	*
Preoperative neurologic status	*	*	*	*	*
Preoperative spinal canal compromise	*	NA	NA	*	*
Preoperative kyphotic angle	*	*	*	NA	*
Preoperative VBH loss	*	NA	NA	*	*
Injury to surgery	*	*	*	NA	*

VBH: vertebral body height. NA: not available.

* Statistically insignificant (p>0.05).

### Kyphotic Angle

Information of kyphotic angle was extracted from three trials (Table 3) [15], [16], [18]. In each study, the difference was not significant between the two groups with regard to postoperative kyphotic angle, correction of kyphotic angle, kyphotic angle at last visit, and lost kyphotic angle. Pooled estimates also revealed no significant difference between the two groups. χ^2^ test showed no evidence of statistical heterogeneity (p>0.1) (Table 4).

**Table 3:** Surgical results of the included studies.

Outcome	Wang 2006	Dai 2009	Jindal 2012	Tezeren 2009
Operative time (min; F vs NF)	194 (224∶162)	127.6(152.0∶102.6)	125.0 (142.4∶107.5)	200.5 (245.2∶155.7)
Blood loss (ml; F vs NF)	442 (572∶303)	367.8(423.7∶310.4)	NA	404.7 (519.0∶290.4)
Hospital stay (d; F vs NF)	15.5 (14.6∶16.6)	12.4 (11.9∶13.0)	NA	10.5 (10.2∶10.8)
Mean postoperative kyphotic angle (°; F vs NF)	2.9 (4.1∶1.6)	0.6 (0.6∶0.5)	4.98 (5.03∶4.93)	NA
Correction of kyphotic angle (°; F vs NF)	15.5 (15.7∶15.3)	17.9 (17.6∶18.2)	12.59 (13.13∶12.05)	NA
Kyphotic angle at last visit (°; F vs NF)	10.7 (11.5∶9.8)	1.5 (1.4∶1.7)	9.51 (10.51∶8.51)	NA
Lost kyphotic angle (°; F vs NF)	7.8 (7.4∶8.3)	1.0 (0.95∶1.04)	4.53 (5.48∶3.58)	NA
Postoperative decreased VBH (%; F vs NF)	13.1 (12.9∶13.3)	NA	NA	8.7 (9.5∶7.8)
Correction of decreased VBH (%; F vs NF)	33.0 (33.9∶32.0)	NA	NA	35.8 (35.5∶36.0)
Decreased VBH at last visit (%; F vs NF)	19.1 (22.3∶15.6)	NA	NA	17.2 (15.2∶19.2)
Lost correction of decreased VBH (%; F vs NF)	6.6 (8.3∶3.6)	NA	NA	8.5 (5.7∶11.4)
Functional outcome assessment at last visit(score; F vs NF)	Greenough low back outcome scale: 66.3 (65.6∶66.9)	Back pain VAS scale: (1.4∶1.5); SF-36 PCS score: (52.3∶53.8); SF-36 MCS score: (65.4∶66.6)	Greenough low back outcome scale: (33.35∶34.92)	Greenough low back outcome scale: (57.8∶55.7)
Fusion status (fusion group)	Fused	Fused	NA	NA
Donor site pain at last visit (fusion group)	7 patient	25 patients; mean VAS score: 5.4	Mean VAS score: 1.56	NA
Implant failure (F vs NF)	8 (5∶3)	0	3 (2∶1)	2 (1∶1)
Neurologic status at last follow-up(Frankel scale; F vs NF)	A (2∶0); B (0∶0); C (0∶0); D (0∶2); E (28∶26)	A (0∶0); B (0∶0); C (0∶0); D (3∶3); E (34∶33)	A (6∶7); B (1∶1); C (2∶5); D (8∶5); E (6∶6)	Neurologic intact

F vs NF: fusion versus nonfusion. NA: not available. VBH: vertebral body height. VAS: visual analog scale. SF-36: short-form-36. PCS: physical component summary. MCS: mental component summary.

**Table 4:** Meta-analysis of the surgical results based on all trials.

Outcome	No. studies	No. patients	H	I^2^	Analysismodel	Pooled estimate (95%confidence intervals)^1^	p
Operative time (min)	4	220	0.02	70%	R	55.04 (32.80, 77.28)	<0.0001
Blood loss (ml)	3	173	0.006	80%	R	189.49 (86.54, 292.45)	0.0003
Hospital stay (d)	3	173	0.83	0%	F	−0.88 (−2.18, 0.42)	0.19
Mean postoperative kyphotic angle (°)	3	178	0.32	13%	F	0.14 (−0.25, 0.53)	0.49
Correction of kyphotic angle (°)	1	58	–	–	F	0.40 (−2.72, 3.52)	0.8
Kyphotic angle at last visit (°)	3	178	0.39	0%	F	−0.20 (−0.85, 0.45)	0.55
Lost kyphotic angle (°)	3	178	0.24	30%	F	−0.05 (−0.63, 0.53)	0.86
Postoperative decreased VBH (%)	2	100	0.46	0%	F	1.14 (−1.32, 3.60)	0.36
Correction of decreased VBH (%)	1	58	–	–	F	1.90 (−3.79, 7.59)	0.51
Decreased VBH at last visit (%)	2	100	0.03	78%	R	1.50 (−8.99, 11.98)	0.78
Lost correction of decreased VBH (%)	1	58	–	–	F	4.70 (1.41, 7.99)	0.005
Functional outcome at last visit^2^	3	147	0.59	0%	F	−0.68 (−3.24, 1.87)	0.6
Implant failure	4	220	0.92	0%	F	1.83 (0.62, 5.40)	0.28
Neurologic status at last follow-up^3^	3	178	–	–	–	–	0.794

VBH: vertebral body height. H: Heterogeneity. R: random effect model. F: fixed effect model.

1 Risk ratios and weighted mean differences were calculated for binary outcomes and continuous outcomes, respectively.

2 Greenough low back outcome scale.

3 Meta-analysis was performed using Wilcoxon rank sum test based on individual patient data.

### Decreased VBH

Relevant data was documented in two articles (Table 3) [15], [17]. There was no difference in mean postoperative decreased VBH and correction of decreased VBH between the two groups. At final follow-up, one study [17] reported similar decreased VBH between the two arms, whereas the other study [15] found there were significant less decreased VBH and less lost correction of decreased VBH in the nonfusion group. The pooled data from the two relevant studies did not reveal any significant difference with regard to decreased VBH after operation and at last follow up (Table 4).

### Function Outcome

The Greenough low back outcome scale was used to assess functional outcome in three papers (Table 3) [15], [17], [18]. There was no significant difference between the fusion and nonfusion groups according to individual and pooled data. Dai and his colleagues [16] applied back pain VAS score and SF-36 score for functional outcome assessment. There was also no significant difference between the two arms at any of the follow-up points. For neurological status at last follow-up, no difference was observed in any of the studies between the two groups. Pooled analysis based on individual data also showed no difference on neurological status between the two arms (Table 4).

### Implant Failure

Data regarding implant failure were provided in four studies (Table 3) [15]–[18]. All studies reported similar implant failure rate between the fusion and nonfusion groups. The overall implant failure rate was 5%. Pooled estimates did not show that the fusion group achieved significantly reduced implant failure rate (RR  = 1.83, 95% CI: 0.62–5.40, p  = 0.28). There was no evidence for significant heterogeneity (I^2^ = 0%; p  = 0.92) (Table 4).

### Operative Time

All four included trials reported significant reduced surgical time in the nonfusion group (Table 3) [15]–[18]. Overall, the weighted mean difference was 55.04 (95% CI: 32.80–77.28, p<0.0001) in favor of the nonfusion group. There was obvious evidence for statistically significant heterogeneity (I^2^ = 70%; p  = 0.02) (Table 4).

### Blood Loss

Three trials investigated perioperative blood loss (Table 3) [15]–[17]. All the trials showed nonfusion significantly reduced perioperative blood loss. Pooling of relevant data also showed statistically significant difference between the two groups (WMD  = 189.5, 95% CI: 86.5–292.5, p<0.0001) (Table 4). Significant heterogeneity was detected (I^2^ = 80%; p  = 0.006).

### Hospital Stay

Details regarding hospital stay were available in three papers (Table 3) [15]–[17]. Statistical heterogeneity was absent in these studies (I^2^ = 0%; p  = 0.83). All three papers showed similar results between the two arms. The pooled estimate revealed statistically insignificant difference (WMD = −0.9, 95% CI: −2.2–0.4, p  = 0.19) (Table 4).

### Subgroup Analysis

In current meta-analysis, three studies which used short segment fixation were RCTs (Table 3) [15], [16], [18]. The study applying long segment fixation was a quasi-RCT [17]. Thus, data were reanalyzed based on two subgroups. For long segment fixation subgroup [17], additional fusion significantly increased the operative time and blood loss. No difference was detected between the fusion and nonfusion groups with regard to other surgical outcomes [17]. For short segment fixation subgroup [15], [16], [18], pooled results did not yield any significant difference compared with the overall effect of all four studies (Table 5).

**Table 5:** Meta-analysis of the surgical results based on trials applying short segment fixation.

Outcome	No. studies	No. patients	H	I^2^	Analysismodel	Pooled estimate (95%confidence intervals)^1^	p
Operative time (min)	3	178	0.17	43%	F	40.72 (30.39, 51.05)	<0.0001
Blood loss (ml)	2	131	0.04	77%	R	174.93 (25.70, 324.17)	0.02
Hospital stay (d)	2	131	0.73	0%	F	−1.27 (−3.30, 0.75)	0.22
Mean postoperative kyphotic angle (°)	3	178	0.32	13%	F	0.14 (−0.25, 0.53)	0.49
Correction of kyphotic angle (°)	1	58	–	–	F	0.40 (−2.72, 3.52)	0.8
Kyphotic angle at last visit (°)	3	178	0.39	0%	F	−0.20 (−0.85, 0.45)	0.55
Lost kyphotic angle (°)	3	178	0.24	30%	F	−0.05 (−0.63, 0.53)	0.86
Postoperative decreased VBH (%)	1	58	–	–	F	−0.40 (−5.15, 4.35)	0.17
Correction of decreased VBH (%)	1	58	–	–	F	1.90 (−3.79, 7.59)	0.51
Decreased VBH at last visit (%)	1	58	–	–	F	6.70 (0.22, 13.18)	0.04
Lost correction of decreased VBH (%)	1	58	–	–	F	4.70 (1.41, 7.99)	0.005
Functional outcome at last visit^2^	2	105	0.96	0%	F	−1.33 (−4.16, 1.51)	0.36
Implant failure	3	178	0.83	0%	F	1.68 (0.53, 5.36)	0.38
Neurologic status at last follow-up^3^	3	178	–	–	–	–	0.794

VBH: vertebral body height. H: Heterogeneity. R: random effect model. F: fixed effect model.

1 Risk ratios and weighted mean differences were calculated for binary outcomes and continuous outcomes, respectively.

2 Greenough low back outcome scale.

3 Meta-analysis was performed using Wilcoxon rank sum test based on individual patient data.

## Discussion

In theory, for surgically treated thoracolumbar burst fractures, additional spinal fusion might increase spinal stability, maintain kyphosis correction, and decrease implant failure rate [3]. However, the findings from our meta-analysis suggested that fusion was not necessary for thoracolumbar burst fractures treated by posterior short or long segment pedicle screw fixation. Both the fusion and nonfusion groups achieved similar radiological and functional outcomes, whereas the nonfusion group had significantly shorter operative time and lower blood loss. There was no significant difference on implant failure rate between the two groups. The clinical significance is that nonfusion avoids bone harvest complications. Furthermore, it facilitates minimally invasive stabilization which reduces the iatrogenic trauma to the posterior structure and promotes healing of the fracture.

Although most studies included in this analysis reported consistent results [15]–[18], the pooled estimates should be explained with caution. The surgical indication, subtype of fracture, location of fracture, and preoperative neurological status were different among the trials. Noteworthy was that over half of the included patients in this study were neurologically intact. A recent meta-analysis [7] revealed there were comparable pain and functional improvement between the operative and nonoperative treatments for thoracolumbar burst fractures without neurologic deficit. Though the operative method was more efficient in residual kyphosis improvement, no association was found between degree of kyphosis and function [7]. Furthermore, surgical treatment was associated with higher complication rates and costs [7]. Therefore, conservative treatment might also be effective for the neurological intact patients in our study.

Posterior short segment pedicle screw fixation has gained increasing popularity for thoracolumbar fractures in the past two decades [3], [4], [8]–[10], [12], [15], [16], [18]. However, reported incidence of implant failure or lost reduction of kyphosis was not low [3], [8]. Tezeren et al. [11] prospectively analyzed the effect of fixed levels on surgical outcomes. They found long segment fixation resulted in better radiological parameters. Nevertheless, the clinical outcomes were similar between the two surgical methods [11]. McCormack et al. [8] proposed a well-known scoring system (the load sharing classification) to predict the screw breakage after posterior short segment pedicle screw fixation. No screw fractures were observed in patients with total scores of less than seven [8]. Altay et al. [13] suggested posterior short segment fixation for neurological intact patients with load sharing score of ≤7 in Magerl Type A3.1 and A3.2, or load sharing score of ≤6 in Magerl Type A3.3. In our meta-analysis, three RCTs [15], [16], [18] applied short segment fixation and the quasi-RCT [17] used long segment fixation. The overall implant failure rates for patients treated with short versus long segment fixation were 6.2% versus 4.8%, respectively. We could not simply attribute the implant failure to fixed levels because it might be affected by other factors such as fracture subtypes, location of fracture, neurological status, surgical techniques, follow up duration, and so on.

Thoracolumbar burst fractures are frequently accompanied by bone fragments retropulsing into the spinal canal. Some studies revealed the phenomenon of spontaneous remodeling of the spinal canal by resorption of the bone fragments [19]–[21] Boerger et al. [20] pointed out that the spinal cord injury was caused by the peak energy that led to the fracture. Thus, decompression seemed not to alter the neurological outcome. Furthermore, laminectomy would destabilize the spine and result in kyphosis [3]. In our review, spinal decompressions were not performed in any of the trials, except for two patients in Dai’s study (one patient in each group undergoing unilateral laminectomy due to fragments of the lamina encroaching into the spinal canal) [16].

Indications for implant removal included malposition screws, instrumental failure, and stubborn infection [22]. However, it remained controversial whether routine implant removal was necessary even when a successful fusion was reached. Reasons for routine implant removal included potential deep late infection, metal toxicity, metal hypersensitivity, corrosion, stress-shielding osteopenia, neoplasia, social culture, and patients’ demands [15], [22], [23]. In Wang’s study [15], removal of implants was only suggested for nonfusion patients, while one third of the patients in the fusion group also requested removal of the implants, either for cultural reasons or back soreness. Routing removal of spinal implant was not performed in the other three trials of our meta-analysis [16]–[18]. Possible reason might be that it added risks such as infection, refracture, nerve injury, and potential large vessel injury [23]–[25].

There are several limitations in this meta-analysis. Firstly, only four small trials were available for inclusion, which means all results and subsequent analysis were based on only 220 patients. Furthermore, several potential biases were detected in the studies analyzed. Therefore, larger randomized controlled trials with high quality are still needed in the future. Secondly, the baseline characteristics were different among the trials. Some confounding factors such as subtype of burst fracture, location of fracture, neurologic status, and surgical technique could have potential affects on clinical and radiological outcomes. In Wang’s study [15], it seemed unusual that the lost correction of vertebral height was higher in the fusion group. This might be explained by the surgical technique applied, as the authors removed the supraspinous, interspinous ligaments and spinous processes before fusion, which could have increased instability of the spine. Thirdly, various outcome measurements were reported in the studies. Standardized and valid items should be used in further studies. Therefore, the clinical applicability of our study should be considered with caution. Despite these limitations, all included trials reported consistent results, and this meta-analysis was based on comparable characteristics between the intervention and control groups. In conclusion, evidence is insufficient to recommend additional fusion for thoracolumbar burst fractures treated by posterior pedicle screw fixation.

## Supporting Information

Figure S1 **Selection of relevant publications, reasons for exclusion.**
(TIF)

Table S1 **Characteristics of the studies included in the meta-analysis.**
(DOC)

Checklist S1 **PRISMA Checklist.**
(DOC)
