# Supplementary material for: Fusion versus Nonfusion for Surgically Treated Thoracolumbar Burst Fractures: A Meta-Analysis
Source: PLoS One. 2013 May 21;8(5):e63995. doi: 10.1371/journal.pone.0063995 (PMC3660321; doi:10.1371/journal.pone.0063995)
Supplement: Table S1 — Characteristics of the studies included in the meta-analysis. (DOC) [file pone.0063995.s002.doc]

**Table S1.** Characteristics of the studies included in the meta-analysis.

| **Characteristic** | **Wang 2006** | **Dai 2009** | **Jindal 2012** | **Tezeren 2009** |
| --- | --- | --- | --- | --- |
| Basic information |  |  |  |  |
| Year of publication | 2006 | 2009 | 2012 | 2009 |
| Study design | RCT | RCT | RCT | Quasi-RCT |
| No. enrolled patients  (F vs NF) | 58 (30:28) | 73 (37:36) | 50 (25:25) | 42 (21:21) |
| No. followed patients  (F vs NF) | 58 (30:28) | 73 (37:36) | 47 (23:24) | 42 (21:21) |
| Follow-up rate  (%; F vs NF) | 100 (100:100) | 100 (100:100) | 94 (92:96) | 100 (100:100) |
| Mean follow-up time  (mo; F vs NF) | 41.0 (39.0:43.1) | 72 (72:72) | 23.9 (24.2:23.6) | 34.6 (33.4:36.0) |
| Mean age  (yr; F vs NF) | 39.8 (39.3:40.9) | 34.6 (35.5:33.7) | 29.4 (29.6:29.3) | 36.2 (38.4:34.1) |
| Gender  (% male; F vs NF) | 71.4 (63.3:82.1) | 76.7 (75.7:77.8) | 63.8 (65.2:62.5) | 71.4 (76.2:66.7) |
| Injury information |  |  |  |  |
| Classification of fracture  (F vs NF) | Denis classification  Type A (11:9)  Type B (19:16)  Type C (0:3) | Denis classification  Type B  McCormack score ≤6 | Classification NA  McCormack score ≤6 | Denis classification  Type A (6:2)  Type B (15:18)  Type C (0:1) |
| Location of fracture  (F vs NF) | T12 (7:2)  L1 (12:9)  L2 (7:17)  L3 (3:0)  L4 (1:0) | T11 (3:2)  T12 (5:6)  L1 (22:19)  L2 (7:9) | T11 (1:1)  T12 (7:6)  L1 (12:12)  L2 (1:4)  L3 (1:1)  L4 (1:0) | T11 (1:2)  T12 (4:6)  L1 (12:11)  L2 (4:2) |
| Preoperative neurologic status  (Frankel scale; F vs NF) | A (2:0)  B (1:0)  C (1:2)  D (6:4)  E (20:22) | A (0:1)  B (0:0)  C (2:3)  D (11:8)  E (24:24) | A (8:10)  B (1:2)  C (8:6)  D (3:2)  E (3:4) | Neurologic intact |
| Mean preoperative spinal canal compromise  (%; F vs NF) | 50.4 (51.9:48.9) | NA | NA | 31.6 (33.0:30.2) |
| Mean preoperative kyphotic angle  (°; F vs NF) | 18.4 (19.8:16.8) | 18.4 (18.2:18.7) | 17.7 (18.46:16.98) | NA |
| Mean preoperative decreased VBH  (%; F vs NF) | 46.1 (46.9:45.3) | NA | NA | 44.4 (45.0:43.8) |
| Injury to surgery  (d; F vs NF) | 4.9 (4.6:5.3) | 3.7 (3.7:3.7) | 8.2 (7.7:8.8) | NA |
| Surgical technique |  |  |  |  |
| No. fixed levels | Short segment | Short segment | Short segment | Long segment |
| Internal fixation use | Pedicle screw fixation | Pedicle screw fixation | Pedicle screw fixation | Pedicle screw fixation + hooks |
| Type of bone graft  (fusion group) | AICBG + local bone | AICBG | AICBG | AICBG |
| Fusion site  (fusion group) | Posterior fusion | Posterolateral fusion | Posterior fusion | Posterior + posterolateral fusion |
| Spinal decompression | No | One in each group | No | No |

RCT: randomized control trial. F vs NF: fusion versus nonfusion. NA: not available. VBH: vertebral body height. AICBG: autogenous iliac crest bone graft.
